# Supplementary material for: Ranking parameters driving siring success during sperm competition in the North African houbara bustard
Source: Commun Biol. 2023 Mar 22;6:305. doi: 10.1038/s42003-023-04698-1 (PMC10033649; doi:10.1038/s42003-023-04698-1)
Supplement: Supplementary file 2 — Supplementary Information [file 42003_2023_4698_MOESM2_ESM.pdf]

Supplementary Information

Ranking parameters driving siring success during sperm competition in the North African houbara bustard

Gabriele Sorci[1*], Hiba Abi Hussein[2], Gwènaëlle Levêque[3], Michel Saint Jalme[4], Frédéric Lacroix[2], Yves Hingrat[2], Loïc Lesobre[2]

[1] Biogéosciences, UMR 6282 CNRS, Université de Bourgogne, 6 boulevard Gabriel, 21000 Dijon, France

[2] Reneco International Wildlife Consultants LLC; Abu Dhabi, PoBox 61741, United Arab Emirates

[3] Emirates Center for Wildlife Propagation, BP 47, route de Midelt, 33250 Missour, Morocco

[4] Centre d'Ecologie et des Sciences de la Conservation, CESCO, Museum National d'Histoire Naturelle, CNRS, Sorbonne Université, Ménagerie le zoo du Jardin des Plantes, 75005 Paris, France

Supplementary Results

Generalized linear mixed model

In addition to the BRT model, we also ran a GLMM with a binomial distribution of errors. The response variable was the fertilization success (0/1). The identities of male and egg were included as crossed intercept random effects. As fixed effects, we included the five most important predictors according to the ranking provided by the BRT model: insemination order, delay between insemination and egg laying, number of sperm in the ejaculate, mass motility index, percentage of days displaying. The model was run using R 3.6.0 (*glmmTMB* package). Multicollinearity was assessed using the *performance* package. All predictors were standardized (mean = 0, SD = 1). The results of the GLMM were in good agreement with the BRT model since the insemination order and the delay between insemination and egg laying had the largest effect sizes (Supplementary Table 1). The slight differences in ranking among the other variables with lower predictive power are not surprising given that the BRT model also includes the non-linear relationship between the predictors and the response variable, and the interactions between different predictors.

Supplementary Table

Table 1. GLMM with a binomial distribution of errors investigating the ranking of predictors of fertilization success. Predictors are ranked according to the absolute value of the parameter estimate. We also report the variance inflation factors (VIFs). N = 901 eggs, 2226 inseminations, 879 males and 599 females.

	Parameter estimate	***Standard Error***	***VIF***
Male insemination order	-1.095	0.101	1.66
Delay between insemination and egg laying	-0.283	0.091	1.65
Mass motility index	0.169	0.057	1.12
Percentage of days displaying	-0.117	0.055	1.01
Number of sperm in the ejaculate	0.064	0.057	1.11

Supplementary Figures

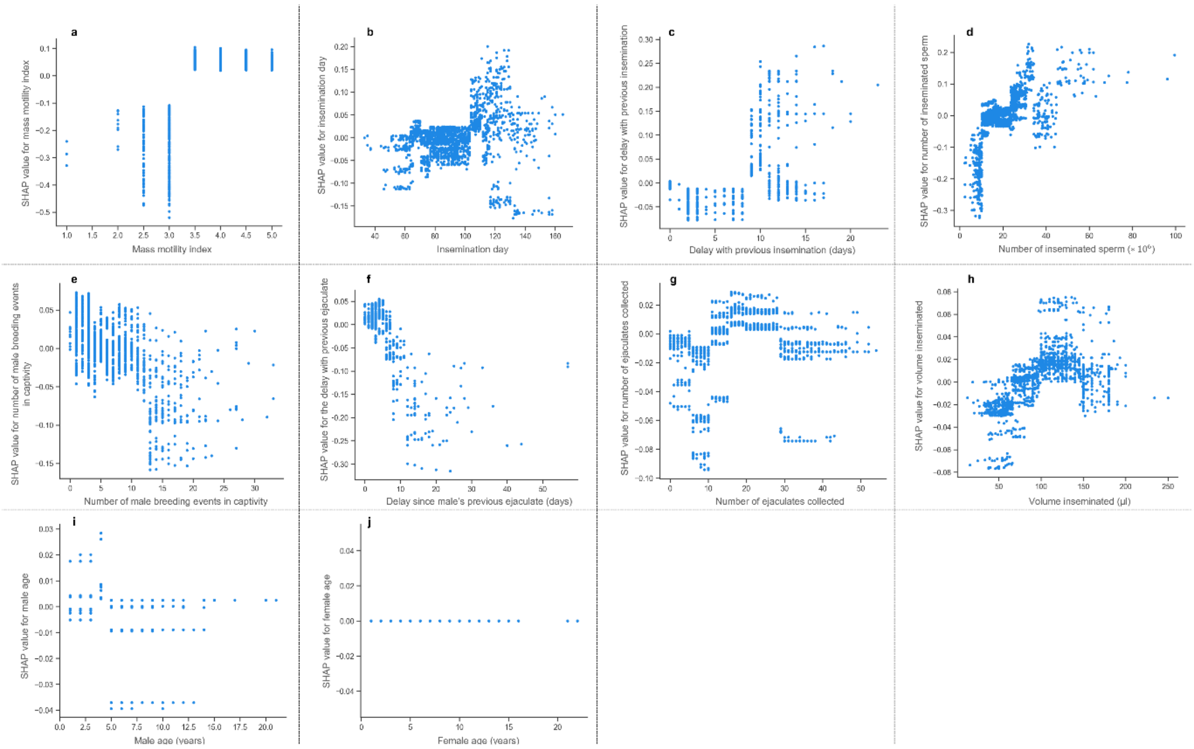

Figure 1. SHAP partial dependence plots of (a) mass motility index; (b) insemination day; (c) delay with previous insemination; (d) number of inseminated sperm; (e) number of male breeding events in captivity; (f) delay since male's previous ejaculate; (g) number of ejaculates collected; (h) volume inseminated; (i) male age; (j) female age. Each dot corresponds to one insemination (N = 2226 inseminations).

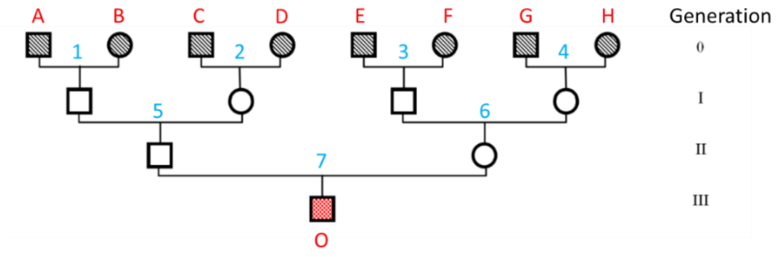

Figure 2. A hypothetical pedigree illustrating the difference between number of generations and number of breeding events. If we assume that individuals A to H are the six founders of a hypothetical captive flock (generation 0), the individual O has generation 3, but it is born after 7 breeding events.

Python script used to run the BRT model

# 1. Importing required libraries

```
import csv
from pickle import TRUE
from typing import Concatenate
import pandas as pd
import matplotlib.pyplot as plt
import gpboost as gpb
import numpy as np
import re
from scipy.__config__ import show
import shap
import sklearn.model_selection
import graphviz
from sklearn.metrics  import matthews_corrcoef,accuracy_score, auc,
roc_curve,  roc_auc_score
from sklearn.model_selection import GroupKFold
```

# 2. Loading data and separation of the features (X), labels (dependent variable, y), and random effects

```
data=pd.read_csv('./Data_Siring_success_2226.csv')
x_data= data.drop(['Unnamed: 0','Male','Female','Fertilisation_success','Egg'
,'number_of_male_generations'], 1)
y_data=np.array(data["Fertilisation_success"])
Egg=np.array(data["Egg"])
Male=np.array(data["Male"])
data_train=gpb.Dataset(x_data,y_data)
group_train=np.column_stack((Male, Egg))
```

# 3. Hyperparameters tuning through grid search that randomly chooses 20 combinations of tuning parameters from the defined grid (param_grid) using 5-fold cross validation

```
gp_model=gpb.GPModel(group_data=group_train,likelihood="bernoulli_logit")
params = {'objective': 'binary', 'verbose': 0, 'is_unbalance':True}
param_grid= {'learning_rate': [0.01,0.005],
            'min_data_in_leaf': [5,10,20,50,100],
                    'max_depth': [3,5,7]}
opt_params_overall = gpb.grid_search_tune_parameters(param_grid=param_grid,
params=params, num_try_random=20, nfold=5, gp_model=gp_model,
use_gp_model_for_validation=True, train_set=data_train, verbose_eval=0,
num_boost_round=2000, early_stopping_rounds=10, seed=1,
metrics='binary_logloss')
```

4. Model training based on the optimal hyperparametres selected in the previous step*

```
bst_params_overall={'objective': 'binary', 'learning_rate': 0.01,
'max_depth':3, 'min_data_in_leaf':50, 'verbose': 0, 'is_unbalance':True}
overall_Model = gpb.train(params=bst_params_overall, train_set=data_train,
            gp_model=gp_model, num_boost_round=1812)
```

# 5. Model interpretation using SHAP library

# SHAP summary plot

```
explainer = shap.TreeExplainer(overall_Model)
shap_values =explainer.shap_values(x_data)
shap.summary_plot(shap_values, x_data)
```

# SHAP absolute mean values extraction

```
def ranking_AbsMean_shap(data_df, shap_values, columns=[]):
    if not columns: columns = data_df.columns.tolist()
    c_loc = []
    for column in columns: c_loc.append(data_df.columns.get_loc(column))
    shap_means = np.abs(shap_values).mean(axis=0)
    features_ranking = pd.DataFrame({'feature': columns, 'mean_shap_value':
shap_means}).sort_values(by='mean_shap_value',ascending=False).reset_index(dro
p=True)
    features_ranking.index += 1
    return features_ranking

ranking_AbsMean_shap (x_data,shap_values, columns=[])
```

# SHAP partial dependency plot

```
shap.dependence_plot(("male_order"),shap_values,x_data,interaction_index=N
one)
```

# 6. Model performance evaluation based on nested Cross validation method

# Function to extract different evaluation metrics based on model prediction

```
def evaluate(model, testing_set_x, testing_set_y,group):
    predictions = model.predict(data=testing_set_x, group_data_pred=group,
            raw_score=False,predict_var=True) ['response_mean']
    accuracy  = accuracy_score(testing_set_y, predictions >= 0.5)
    roc_auc   = roc_auc_score(testing_set_y, predictions)
    mcc = matthews_corrcoef(testing_set_y, predictions >= 0.5)
    result = pd.DataFrame([[mcc, accuracy, roc_auc]],
columns=['mcc','Accuracy','ROC_auc'])
    return(result)
```

# Data splitting (number of folds=5), nested cross validation for hyperparameters tuning and performance evaluation at each fold

```
eval=dict()
```

```
Train_indexes=dict()
Test_indexes=dict()
Training_ind_df=pd.DataFrame(index=range(1781)  )
Test_ind_df=pd.DataFrame(index=range(446)  )
gkf = list (GroupKFold( n_splits=5).split(X=x_data,y=y_data,groups=Egg))
k=0
for train_index, test_index in gkf:
    Train_indexes[k]=train_index
    Test_indexes[k]=test_index
    X_train, X_test = x_data.iloc[train_index], x_data.iloc[test_index]
    y_train, y_test = y_data[train_index], y_data[test_index]
    group_train=np.column_stack((Male[train_index], Egg[train_index]))
    group_test=np.column_stack((Male[test_index], Egg[test_index]))
    data_train=gpb.Dataset(X_train,y_train)
    gp_model=gpb.GPModel(group_data=group_train, likelihood="bernoulli_logit")
    opt_params = gpb.grid_search_tune_parameters( param_grid=param_grid,
params=params, num_try_random=20, nfold=5, gp_model=gp_model,
use_gp_model_for_validation=True, train_set=data_train, verbose_eval=0,
num_boost_round=2000, early_stopping_rounds=10, seed=1,
metrics='binary_logloss')
    params=opt_params['best_params']
    num_boost_round=opt_params["best_iter"]
    best_mod = gpb.train(params=params,
                    train_set=data_train,
                    gp_model=gp_model,
                    num_boost_round=num_boost_round)
    path='./best_mod_5FCV_K_'+str(k)+'.json'
    best_mod.save_model(path)
    eval[k]=evaluate(best_mod,X_test,y_test,group_test)
    k=k+1
```
